# Treatment Failure after Multiple Courses of Triclabendazole among Patients with Fascioliasis in Cusco, Peru: A Case Series

**DOI:** 10.1371/journal.pntd.0004361

**Published:** 2016-01-25

**Authors:** Miguel M. Cabada, Martha Lopez, Maria Cruz, Jennifer R. Delgado, Virginia Hill, A. Clinton White

**Affiliations:** 1 Department of Internal Medicine, University of Texas Medical Branch, Galveston, Texas, United States of America; 2 Sede Cusco—Instituto de Medicina Tropical Alexander Von Humboldt, Universidad Peruana Cayetano Heredia, Lima, Peru; 3 Hospital Nivel IV Adolfo Guevara Velasco ESSALUD, Cusco, Peru; 4 College of Natural Sciences, University of Texas at Austin, Austin, Texas, United States of America; Universidad Peruana Cayetano Heredia, PERU

## Abstract

Triclabendazole is reported to be highly effective in treatment of human fascioliasis. We present 7 of 19 selected cases of human fascioliasis referred to our center in the Cusco region of Peru that failed to respond to triclabendazole. These were mostly symptomatic adults of both sexes that continued passing *Fasciola* eggs in the stool despite multiple treatments with 2 doses of triclabendazole at 10 mg/kg per dose. We documented the presence of eggs by rapid sedimentation and Kato Katz tests after each treatment course. We found that repeated triclabendazole courses were not effective against fascioliasis in this group of people. These findings suggest that resistance to triclabendazole may be an emerging problem in the Andes.

## Author Summary

Fascioliasis is a zoonotic food borne trematode infection with a wide distribution. The complex epidemiology of this infection makes control efforts difficult. The paucity of drugs available for treatment may further hinder their success. Triclabendazole, the only first line drug for Fasciola, has been used for many years in the livestock industry. Resistant livestock Fasciola infections have emerged in developing and developed countries. However, most human trials report triclabendazole efficacies close to 100% after a few doses. Only a few cases of triclabendazole treatment failure have been published. We document 7 patients infected with *Fasciola hepatica* in Cusco–Peru that failed several treatment courses with triclabendazole. This raises concerns regarding preparedness to address resistant parasite infections and calls for more research to find new medications and tools to evaluate resistance.

## Introduction

Fascioliasis is a worldwide zoonotic infection caused by the trematode parasite *Fasciola hepatica*. Livestock infection causes large economic losses in developed and developing countries.[1] Even in some wealthy countries, up to 50% of the dairy and meat herds may be infected; but data from resource-poor countries are limited.[2–4] Heavily infected cattle have significantly decreased milk (≥ 1.5 L daily) and meat (≥ 3 kg) production.[5,6] Human infection has been reported in more than 70 countries, but the highest burden occurs in the Andes and parts of the Middle East.[7] School-age children have the highest prevalence of fascioliasis and bear most of its severe consequences. Lopez et al. described a threefold increase in anemia risk among children with fascioliasis compared with children without infection.[8] Significant weight loss during the acute and chronic infections has been described in other studies.[9,10] Thus, the long term effects of fascioliasis complications have motivated significant efforts to tackle livestock and human infection.

Triclabendazole is the most effective drug for fascioliasis based on safety and cure rates reported in small mostly uncontrolled studies.[11] Mass treatment with triclabendazole has been proposed as a strategy to control fascioliasis in livestock and humans. In developed countries cattle and sheep herds are treated with triclabendazole under professional supervision. However, in resource-poor countries, mass livestock treatment is often inconsistent.[12] Mass treatment and inconsistent dosing of triclabendazole may select resistant parasites.[13] The emergence of triclabendazole resistance has been described among sheep and cattle herds in Scotland, Northern Ireland, and Australia and has been associated with decreased beef and dairy production.[12,14] Increasing resistance has also been reported in Cajamarca, Peru, where only 31% of cattle treated with 12 mg/kg of triclabendazole were cured after 14 days.[15] Triclabendazole resistance in humans has only rarely been noted.[16,17] Reports of resistance are of concern given that triclabendazole is the only highly effective treatment available. In this report, we describe 7 patients with fascioliasis with persistent infection despite multiple treatment courses with triclabendazole.

## Methods

The Cusco region in the south highlands of Peru is an endemic area for fascioliasis. In rural areas of this region the prevalence of *Fasciola hepatica* infection among children is 11%.[8] The Universidad Peruana Cayetano Heredia and University of Texas Medical Branch Collaborative Research Center in Cusco is a referral center for research and management of *Fasciola* infection. Patients referred to us with diagnosed or suspected fascioliasis are evaluated with up to three Lumbreras rapid sedimentation and Kato Katz stool tests. Subjects with negative stool tests and significant eosinophilia are evaluated with Fas2 ELISA for serum antibodies against *Fasciola hepatica*. Except when noted, treatment courses for patients with stool or serologic evidence of fascioliasis consisted of 2 doses of triclabendazole at 10 mg/kg every 12 hours preceded by a meal rich in fat. All subjects received counseling on avoidance of vegetables that might put them at risk for reinfection. Treatment response was assessed with Lumbreras rapid sedimentation and Kato Katz stool tests between 1 and 3 months after treatment.[18,19] Those found to remain infected received repeated courses of triclabendazole.

### Cases report

**Fig 1:**
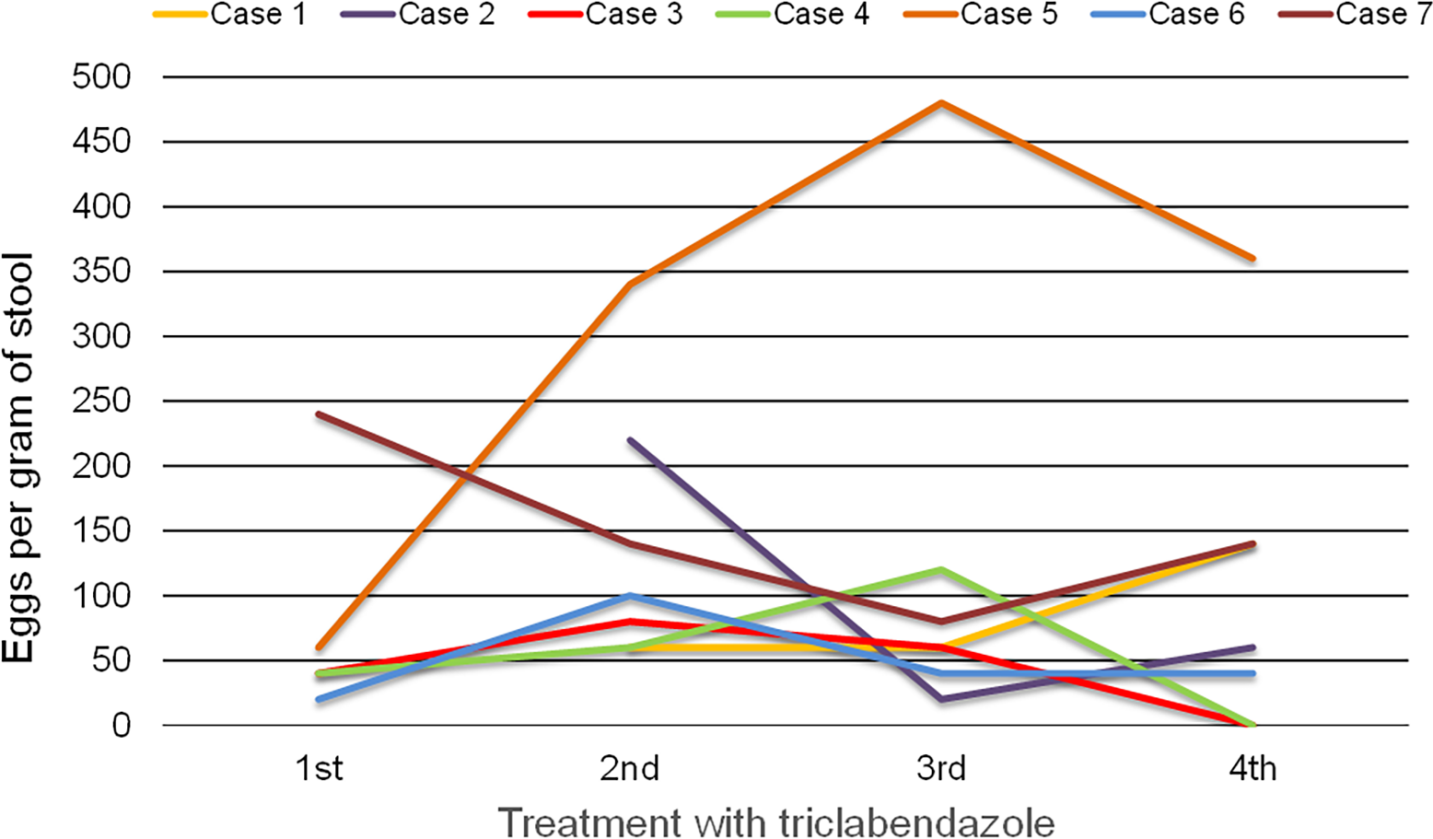
*F*. *hepatica* egg counts 1 to 3 months after triclabendazole treatment.

Between January 2014 and April 2015, 7 out of 19 patients with *Fasciola hepatica* infection referred to our center for evaluation and treatment failed to respond to multiple courses of triclabendazole (Egaten 250 mg tablets, Novartis Pharma AG, Basel, Switzerland, expiration date December 2015) 2 doses at 10 mg/d every 12 h with a fatty meal. Three of these were males, 2 were younger than 18 years old, and all but one were born in Cusco City. Four patients had acute presentations with delayed diagnosis, severe symptoms requiring prolonged hospital admissions for hypereosinophilia. All patients had a history of eating fresh watercress and other green leafy vegetables and self-medicated at least once with triclabendazole for veterinary use without response. In an attempt to improve their clinical response, all were prescribed triclabendazole and nitazoxanide (Colufase, Roemmers SA, Lima, Peru) 500mg PO every 12 hours for 7 days in combination after failing courses of treatment with triclabendazole monotherapy. Only one patient had consecutive negative stool tests and was deemed cured. The characteristics of the patients are shown in Table 1. The cases and their clinical course are briefly described below. Fig 1 shows the egg counts 1 to 3 months after each treatment course with triclabendazole.

**Table 1:** Characteristics of the patients with fascioliasis that failed triclabendazole treatment.

Case	Age	Sex	Clinical presentation	Eosinophilia	Times treated*	Days of admission	Complications
1	51	F	Abdominal pain, weight loss, fever	Yes	4	30	No
2	36	F	Abdominal pain, weight loss, multiple liver lesions on CT scan	Yes	3	15	No
3	43	F	Asymptomatic	No	3	None	Subcutaneous nodule and hypereosinophilia
4	42	M	Abdominal pain, weight loss, fever, rash, multiple liver lesions on CT scan	Yes	4	10	No
5	15	M	Abdominal pain, pleural effusion, ascitis, fever, multiple liver lesions on CT scan	Yes	4	60	No
6	12	M	Asymptomatic	No	4	None	Mild abdominal pain and vomiting
7	36	F	Asymtomatic	No	4	No	Severe abdominal pain and vomiting

* Times treated: Number of times the patient was prescribed treatment. The use of veterinary formulation of triclabendazole is not included as it was impossible to ascertain the dose received.

Case 1 was a 51 years old female farmer with 16 kg weight loss, right upper quadrant abdominal pain, night sweats, anorexia, malaise, fever, and hypereosinophilia. She was first treated with a single dose of 10 mg/kg triclabendazole with improvement of symptoms. However, after 8 months her symptoms returned and she was again noted to be shedding *Fasciola* eggs. The patient was treated 2 additional times with triclabendazole (each with 2 doses of 10 mg/kg every 12 hours) and then with 2 doses of triclabendazole 10 mg/kg every 12 hours followed by nitaxozanide 500 mg every 12 hours for 7 days with improvement of symptoms. However, continued to pass *Fasciola* eggs on stool tests.

Case 2 was a 36 years old female with right upper quadrant abdominal pain, jaundice, severe joint pain, fatigue, 8 kg weight loss, and hypereosinophilia. Her stool tests and Fas-2 ELISA were positive for *Fasciola*. After failing a treatment course with triclabendazole (2 doses of 10 mg/kg every 12 hours), she was referred to our center. Over 10 months she received 1 additional course of triclabendazole treatment (2 doses of 10 mg/kg every 12 hours), a course of 3 doses of triclabendazole 10 mg/kg every 12 hours, and a course of 2 doses of triclabendazole 10 mg/kg every 12 hours followed by nitazoxanide (500 mg every 12 hours for 7 days) with marked improvement of symptoms but persistence of *Fasciola* eggs in the stools.

Case 3 was a 43 years old female who was asymptomatic. She was tested for *Fasciola hepatica* ova after her husband (case 4) was diagnosed with the infection. Both Fas2 ELISA and stool tests were positive for *Fasciola* infection. She failed a course of triclabendazole (2 doses of 10 mg/kg every 12 hours) and was prescribed 2 doses of triclabendazole 10 mg/kg every 12 hours followed by nitaxozanide 500 mg every 12 hours for 7 days after which she developed intrahepatic bile obstruction with removal of 3 adult *Fasciola* by endoscopic retrograde cholangiopancreatography. Also a migratory subcutaneous nodule due to *Fasciola* developed despite self-medication with a veterinary formulation of triclabendazole in 2 occasions. She was prescribed triclabendazole (2 doses of 10 mg/kg every 12 hours) with negative stool tests for *Fasciola* at 5–6 weeks follow up.

Case 4 is a 42 years old male born in the jungle of Cusco state married with case 3. He was admitted to the hospital with fever, diffuse abdominal pain, cough, severe fatigue, 5 kg weight loss, rash in the lower extremities and buttocks, and hypereosinophilia (eosinophil count > 30,000/dL). Lumbreras rapid sedimentation and Fas2 ELISA tests were positive for *Fasciola hepatica* infection. A few days after receiving triclabendazole treatment (2 doses of 10 mg/kg every 12 hours) his symptoms disappeared, but continue passing *Fasciola* ova. He was prescribed 2 additional treatment courses with triclabendazole (2 doses of 10 mg/kg every 12 hours) followed by a course of 2 doses of triclabendazole 10 mg/kg every 12 hours combined with nitaxozanide 500 mg every 12 hours for 7 days and 2 courses of triclabendazole veterinary formulation turning his Kato Katz tests negative. However, the Lumbreras rapid sedimentation test has remained positive.

Case 5 was a 15 years old male who presented with epigastric pain, fever, shortness of breath, chest pain, 5 kg weight loss, hypereosinophilia, ascites, and pleural effusion. The stool tests for *Fasciola* were initially negative, but the Fas2 ELISA was positive. He was treated with a single dose of veterinary formulation triclabendazole with partial improvement of symptoms. His follow up stool tests were positive and remained positive since then despite 3 courses of triclabendazole (2 doses of 10 mg/kg every 12 hours each), and a course of 2 doses of triclabendazole 10 mg/kg every 12 hours in combination with nitaxozanide 500 mg every 12 hours for 7 days.

Case 6 was a 12 years old male brother of case 5 diagnosed with asymptomatic *F*. *hepatica* infection. He failed the initial treatment with a single dose of triclabendazole. He was prescribed and failed 2 additional triclabendazole courses (2 doses of 10 mg/kg every 12 hours). He was subsequently treated with 2 doses of triclabendazole 10 mg/kg every 12 hours followed by nitaxozanide 500 mg every 12 hours for 7 days but his stool tests have remained positive for *Fasciola* eggs.

Case 7 was a 36 years old woman mother of case 5 diagnosed with chronic *F*. *hepatica* infection. She received 3 courses of triclabendazole (2 doses of 10 mg/kg every 12 hours) followed by a course of 2 doses of triclabendazole 10 mg/kg every 12 hours combined with nitaxozanide 500 mg every 12 hours for 7 days with persistently positive stool tests.

### Ethics statement

The study was reviewed by the Institutional Ethics Committee from Universidad Peruana Cayetano Heredia in Lima, Peru. Written informed consent was obtained from the subjects.

## Results/Discussion

Although triclabendazole resistance in veterinary medicine is well known, resistant human infections have only rarely been reported. In this manuscript, we report 7 cases of *Fasciola hepatica* infection that failed to respond to multiple treatment courses with recommended doses of triclabendazole. Two other case reports of failure of triclabendazole treatment for fascioliasis have been published. In 2012, Winkelhagen et al. from the Netherlands reported a single case of multiple treatment failures with triclabendazole and nitazoxanide.[16] In 2014, Gil et al. reported 4 cases of triclabendazole failure in Chile.[17] However, in 3 of those cases the timeline between symptoms, treatment, and evaluation of response suggest reinfection rather than treatment failures. Of note, none of the reported cases had quantitative tests to evaluate the response of egg burden to treatment. Most of our patients had low egg burdens and the egg counts did not showed significant reductions after treatment.

The microscopic detection of *Fasciola* eggs in human stool after the ingestion of metacercaria takes approximately 12 weeks. Our cases were followed up and tested for cure between 1 and 3 months after treatment with triclabendazole. This approach to monitoring was chosen to distinguish the treatment response from reinfection. Failure of anthelmintic treatment may be due to a number of factors. Quality control of the medications is essential. In these cases, all treatment failures received Egaten 250 mg tablets (Novartis Pharma AG, Basel, Switzerland) stored according to the manufacturer recommendations with an expiration date in December 2015 (well after treatment). Treatment failure may also result from inadequate drug absorption. Food has significant effects on the absorption of triclabendazole. It is recommended that patients with fascioliasis ingest a fatty meal before each triclabendazole dose to increase medication absorption in the intestine, as was done in all of our cases. The impact in cure rates of not following this recommendation has not been studied. However, uncontrolled clinical trials with single dose triclabendazole report efficacies around 90% despite absence of fat ingestion before treatment.[11] Reduced triclabendazole conversion to triclabendazole sulfoxide and triclabendazole sulfone in the presence of severe liver impairment have been proposed as a cause of treatment failure.[20] All our cases had relatively low *Fasciola* egg counts suggesting mild infections and probably minimal liver damage. Of note, most of our patients presented with symptoms of acute *Fasciola* infection. Whether this was associated with the initial treatment failure cannot be ascertained. In early stages of infection with *Schistosoma sp*. the parasite has reduced susceptibility to praziquantel as demonstrated *in vitro* and *in vivo*.[20,21] Some *in vitro* studies with *Fasciola hepatica* infection suggest reduced triclabendazole susceptibility among juvenile parasites compared to adults.[22] This has not been rigorously documented in case series of patients with acute infections.[23,24] Marcos et al. reported the resolution of eosinophilia after a single dose of triclabendazole in 10 patients with acute *Fasciola* infection. However, the authors failed to report the absence of eggs in the stools during the follow up.[25] Thus, the clinical evidence gathered suggests the presence of triclabendazole resistant *Fasciola hepatica* infection in our cases.

The mechanisms by which the fluke can become resistant to triclabendazole remain to be elucidated. The β-tubulin gene mutations that cause benzimidazole resistance in nematode parasites does not seem to explain triclabendazole resistance in *Fasciola hepatica*.[26] Changes in drug uptake and parasite drug metabolism seem to play a bigger role. The uptake of the drug by the fluke is influenced by a P-glycoprotein linked efflux pump. Experiments have shown that inhibition of this pump leads to potentiation of triclabendazole activity.[27] In addition, triclabendazole resistant flukes have been shown to metabolize triclabendazole sulfoxide to sulfone to a greater extent than susceptible flukes.[28]. Thus, the combined effect of reduced drug uptake and more active drug metabolism could reduce the effective concentrations of triclabendazole.

Triclabendazole has been used in livestock to treat Fasciola for many years. Inconsistent dosing and schedules have led to widespread resistance in cattle rearing areas in the last decade. Human infections with triclabendazole resistant Fasciola in areas with zoonotic transmission is a potential problem. In contrast to veterinary medicine in which other treatment options for Fasciola exist, in humans triclabendazole is the only first line medication with reported high efficacy. Thus, the emergence of triclabendazole resistance in fascioliasis among humans is an important clinical and public health concern as no alternative drugs are available to treat the infection. In our case series, subjects were treated with nitaxozanide (500 mg twice daily for 7 days) after 2 doses of triclabendazole. This approach was based on a double blind placebo controlled trial of nitazoxanide for the treatment of fascioliasis. Although the trials showed limited efficacy in children (40%), the efficacy was slightly higher in adults (60%).[29,30] The development of biliary colic in some of the cases could have suggested response to the medication, but none were cured by combination treatment.

This study has some limitations that made difficult the assessment of resistance. Most of the information was collected retrospectively and the number of cases was small. We were not able to recover live flukes from the subjects after treatment or generate metacercariae from the eggs collected for susceptibility testing *in vitro*. Nevertheless, our clinical observations suggest the presence of triclabendazole resistant *Fasciola* infections in a selected group of patients from Cusco. Resistant infection in livestock has already been reported in the northern highlands of Peru. Although, this report does not reflect in any way the community prevalence of triclabendazole resistance among humans in Cusco, triclabendazole resistance appears to be an emerging problem deserving attention in Peru and probably other highly endemic areas. Research on new drugs and methods to evaluate drug resistance is urgently needed to control *Fasciola*.
